# Increasing the Awareness of Stroke Among Canadian Women

**DOI:** 10.7759/cureus.23159

**Published:** 2022-03-14

**Authors:** Ibraheem O Alimi, Arielle Archibald, Odera Nwankwo, Deepak Palanichami, Tolulope Oladimeji, Emmanuel Keku

**Affiliations:** 1 Public Health and Preventive Medicine, St. George's University, School of Medicine, St. George's, GRD; 2 Medicine, St. George's University, School of Medicine, St. George's, GRD

**Keywords:** family medicine/general practice, neurology, primary prevention, health promotion, health policy, epidemiology, public health, canada, women, stroke

## Abstract

Previous studies have shown that there is increased mortality of cerebrovascular diseases such as stroke among Canadian women. The morbidity of stroke is also higher among Canadian women because they are less likely to recover from stroke, and they generally tend to have a greater disability from a stroke when compared to men. In order to help minimize these issues, six interventional strategies were evaluated using four criteria: 1) the evidence-based criterion, 2) the socioeconomic-based criterion, 3) the ethics-based criterion, and 4) the sustainable-based criterion. Upon analysis, two alternative interventional strategies were recommended: increased public awareness of stroke symptoms and increased public awareness of stroke risk factors among Canadian women.

## Introduction

Epidemiology of stroke among women in Canada

As of 2020, cerebrovascular diseases such as stroke are the fifth leading cause of death in Canada, with cancer and cardiovascular diseases taking the first and second position, respectively. In 2016, 13,551 mortalities occurred due to stroke, accounting for approximately 6.5% of all mortalities [1]. According to the 2018 Stroke Report released by the Heart and Stroke Foundation of Canada, approximately 59% of all death from stroke in Canada are women, whereas men make up 41% of all stroke mortalities. Regarding stroke incidence in Canada, there are approximately 62,000 new cases of stroke occurring yearly. Approximately 49% of the new cases (around 30,200) occur in women. The prevalence of stroke among women is also higher in Canada when compared to men. As of 2018, approximately 405,000 people were living with stroke in Canada. Out of this total, approximately 214,000 are women, whereas 191,000 are men [2].

Women are also less likely to recover from stroke, and they generally tend to have greater morbidity and disability from a stroke when compared to men [3,4]. The outcomes are also generally worse, with twice as many Canadian women than men attending long-term care after suffering from a stroke. Approximately 46% of Canadian women participate in rehabilitation [2]. As a result of this, Canadian women generally recover slowly from a stroke. Older Canadian women, most especially those above the age of 80 years old, have a significantly increased stroke incidence [4,5]. Due to the longer life expectancy in women and the significantly higher incidence at old age, women are disproportionally affected by a stroke throughout their lifetime [3]. Some of the risk factors of having a stroke in women include hypertension, atrial fibrillation, diabetes, high cholesterol level, obesity, unhealthy diet, lack of exercise, stress, smoking, alcohol abuse, and drug abuse [2]. All these risk factors are preventable, and they also pertain to men.

Public perception of stroke among women in Canada

Similar to the low public awareness of cardiovascular diseases in women, there is an even lower public awareness and recognition of the higher prevalence, incidence, mortality, and morbidity of stroke among Canadian women [2]. One of the reasons for this is because early signs of stroke such as transient ischemic attacks (TIAs); brief episodes and symptoms of stroke that normally resolves themselves within minutes or hours, tend to be subtler and more unnoticed in women and, in some cases, strokes are misdiagnosed as TIAs in women [3,4]. Stroke is also unnoticed in women because there is more media coverage of stroke in men than women, and it is mostly associated with men due to the media and history of prominent men such as Franklin Roosevelt, Woodrow Wilson, and Richard Nixon [6]. Public awareness and medical response to stroke within the first few hours after having a stroke are critical for survival, morbidity, and recovery. The more public awareness of stroke in women, the faster the symptoms of a stroke are recognized so that stroke patients can be taken to the hospital for timely intervention. This will also significantly improve their chances of survival, recovery as well as the chances of not having a permanent disability.

There is also a low public awareness and recognition of stroke symptoms among Canadian women. According to a previous poll by the Heart and Stroke Foundation of Canada, only 60% of women know what a stroke is [2]. Out of this 60%, 36% do not know any of the face, arm, speech, and time (FAST) approach for recognizing symptoms of stroke, and only 8% know all the FAST symptoms of stroke, which includes checking for stroke symptoms. The public’s lack of awareness and recognition of stroke symptoms in women is most especially more pronounced in young Canadian women that are experiencing a stroke. Young women take the longest average time (nine hours) to arrive at the hospital after having a stroke. For older women, the average is slightly better at 7.5 hours. Research focusing on the increased risk of stroke in women, especially older women, is limited and under-represented in stroke research. Compared to heart disease research, research on cerebrovascular diseases such as stroke are relatively new, and there is still much to be revealed about the biological differences between the male and female brain that might also contribute to the differences in the prevalence, incidence, mortality, morbidity, and recovery of stroke among Canadian men and women [2].

Many women are also unaware of their risk factors for stroke, as shown by a previous poll by the Heart and Stroke Foundation of Canada, which revealed that 70% of women do not know any risk factors for stroke, and only about 25% of women identified hypertension as a risk factor for stroke. Less than 1% of women identified atrial fibrillation as a risk factor which is the second most important risk factor for stroke in women after hypertension [2].

Ethics of stroke among women in Canada

Women are biologically different from men, and as they develop over time, they biologically have a disproportionately increased risk of developing stroke at certain points during their lifetime. Women are at an increased risk of developing stroke during pregnancy, after menopause as well as during old age, all of which present additional risk factors that are unpreventable and absent in men. The significantly increased risk of stroke in women during delicate and vulnerable periods such as pregnancy and old age presents ethical reasons for more public awareness of stroke in women [3,4].

The financial burden of stroke among women in Canada

The cost of acute stroke care is significantly higher in women when compared to men. Previous studies have shown that even though the average cost of acute stroke care per patient was $27,500 Canadian dollars (CAD), stroke care in Canadian men cost significantly less when compared to Canadian women ($23,000 CAD/patient in men versus $32,000 CAD/patient in women) [7]. One of the main reasons for this is because women were generally hospitalized longer than men due to stroke complications [3].

Overview of the Public Health Agency of Canada’s surveillance of stroke among women

The Public Health Agency of Canada (PHAC) is the Canadian federal institution that “empowers Canadians to improve their health. In partnership with others, its activities focus on preventing disease and injuries, promoting good physical and mental health, and providing information to support informed decision-making. It values scientific excellence and provides national leadership in response to public health threats” [8].

The PHAC releases a yearly report called the Canadian Chronic Disease Surveillance System (CCDSS). The CCDSS is a collaborative effort between the federal and provincial governments that “enhances the scope of data on chronic diseases in Canada and supports the planning of health resources and the development of health policies and programs” [9]. Currently, there are 20 chronic diseases on the PHAC CCDSS. Stroke is included in this list under the category of cardiovascular diseases. The most current CCDSS report on stroke was released in September 2017, and it summarizes national surveillance data on the incidence, prevalence, and mortality of stroke in Canada from 2003 to 2013. According to the report, approximately 741,800 stroke survivors experienced a stroke as of 2012/2013, which is significantly higher than the number of stroke survivors who experienced stroke 10 years ago in 2003/2004, which was approximately 526,200. The incidence of stroke was slightly higher in males across all age groups except for those above the age of 80 years old [5].

Although chronic diseases such as stroke are recognized as public health issues by the Public Health Agency of Canada (PHAC), there is very little awareness and health promotion released by the PHAC regarding the increased incidence, prevalence, morbidity, and mortality of stroke among women in Canada. The most recent surveillance report regarding this issue that was released by the PHAC in 2017 shows that the PHAC policies are significantly lacking when it comes to health promotion on the issue of stroke among women in Canada [5].

## Materials and methods

This article utilizes four criteria that can be used to evaluate the status quo as well as the alternative interventional strategies. These include 1) the evidence-based criteria, 2) the socioeconomic-based criteria, 3) the ethics-based criteria, and 4) the sustainable-based criteria. These evaluation criteria are adapted from the research methodology typically utilized by brief policy studies and program evaluation standards [10,11]. 

Evidence-based criterion

An evidence-based criterion will be the main criteria for evaluating most of the arguments for and against the alternatives. The reason for this is that the main issue with regards to stroke among women is the fact that they suffer from an increased incidence (at old age), prevalence, morbidity, and mortality of stroke among women in Canada. All of these can potentially be reduced by applying specific alternatives.

Socioeconomic-based criterion

A socioeconomic-based criterion would be considered when analyzing the arguments supporting or opposing an alternative. This ensures that the alternative is socially and economically feasible for the government to achieve. There are limited resources available for the Canadian provincial/territorial government to deliver universal health insurance to Canadians, which is why it is important that the proposed alternative fit within the government’s proposed budget. This is also important because acute stroke care for women is generally more expensive than for men [7].

Ethics-based criterion

The ethics-based criterion examines gender equality and fairness of the alternative intervention. When compared to men, women have an increased risk of getting stroke during vulnerable periods such as pregnancy and old age. This evaluation criterion also advocates for equal awareness, recognition, treatment, and rehabilitation of stroke among men and women.

Sustainability-based criterion

The sustainability-based criterion can be used to evaluate whether an alternative can be sustainably developed and maintained over a long period of time. Alternatives that fulfil the evidence-based and socio-economic based criteria are also likely to fulfill this criterion. The alternative should also fulfill specific United Nations sustainable development goals (SDG) 2030 that are related to the issue of stroke among women in Canada. This includes SDG 3 (“Ensure healthy lives and promote well-being for all at all ages”), SDG 5 (“Achieve gender equality and empower all women and girls”), SDG 10 (“Reduce inequality within and among countries”) and SDG 16 (“Promote peaceful and inclusive societies for sustainable development, provide access to justice for all and build effective, accountable and inclusive institutions at all levels”) [12].

Status quo of stroke among Canadian women

The first alternative is to maintain the status quo and leave things the way they currently are without classifying the increased incidence, prevalence, morbidity, and mortality of stroke among Canadian women as a public health problem that requires improved health polices by the government and more health promotion by the PHAC to help minimize these problems.

Alternative 1: increased public awareness of stroke symptoms among Canadian women

This alternative promotes better and more up-to-date surveillance by the PHAC on stroke among Canadian women. This will assist the PHAC in designing strategies for health promotion in order to improve the public’s awareness and recognition of stroke among Canadian women. The greater the public’s awareness of stroke symptoms in women, the faster the signs can be recognized so that the victim can be taken to the hospital as quickly as possible for acute management. This will significantly reduce the chances of having a permanent disability as well as improve their chances of survival and recovery.

Alternative 2: increased public awareness of stroke risk factors among Canadian women

This alternative encourages more women to be aware of their risk factors for having stroke. Many women are unaware of their risk factors for having stroke which makes them even more susceptible to stroke. Many of these risk factors can be prevented if the awareness of the risk factors for stroke were adequate.

Alternative 3: improved stroke diagnosis among Canadian women

Stroke is often misdiagnosed as transient ischemic attacks in females and in some cases some early signs of stroke may go unnoticed due to the benign and subtle nature [3,4]. Improved stroke diagnosis in women can be achieved with better access to CT as well as MRI imaging [2]. MRI with diffusion imaging is recommended in cases experiencing TIA symptoms. The detection of acute ischemic injury on early MRI, would be considered stroke even though symptoms may be subtle or resembling TIA [13]. Earlier stroke detection by using either MRI or CT imaging can then allow for earlier treatment interventions.

Alternative 4: improved stroke recovery and rehabilitation among Canadian women

Women with the diagnosis of stroke tend to have more morbidity as well as poorer recovery and rehabilitation when compared to men [3,4]. Unlike men, women generally lack the motivation and support they need to recover and participate in rehabilitation. Men with stroke generally have their family such as wives, mothers, and daughters to support and motivate them during recovery and rehabilitation from stroke whereas on the other hand, women with stroke tend to be lonelier [2]. Therefore, more support and motivation needs to be provided for women during stroke rehabilitation.

Alternative 5: increased research on stroke among Canadian women

Stroke research focusing on the increased risk of stroke in women, especially older women, is limited. In addition to this, the majority of participants in stroke epidemiological research tend to be males (57% in men vs. 43% in women [2]). There needs to be more research on women and more representation of women in stroke research.

## Results

Status quo of stroke among Canadian women

Arguments For

Evidence-based criterion: The main argument for keeping the status quo is with regards to the incidence of stroke between men and women. Although Canadian women have an increased incidence of stroke above the age of 80, men have a higher incidence in all other age groups. Although the prevalence and morbidity of stroke may be higher in women, they generally have a longer life expectancy which provides more time for recovery when compared to men (Table 1) [5].

**Table 1:** Summary of the analysis and evaluation criteria supporting the arguments for each alternative and the status quo "Yes" refers to an interventional strategy supported by the arguments for each evaluation criterion. "No" refers to an interventional strategy that is not supported by the arguments for each evaluation criterion.

Alternatives	Evidence-based criterion	Socioeconomic-based criterion	Ethics-based criterion	Sustainable-based criterion
A) Status quo	Yes	No	No	No
B) Alternative 1	Yes	Yes	Yes	Yes
C) Alternative 2	Yes	Yes	Yes	Yes
D) Alternative 3	Yes	No	Yes	Yes
E) Alternative 4	Yes	No	Yes	Yes
F) Alternative 5	Yes	No	Yes	Yes

Arguments Against

Evidence-based criterion: More women die from stroke every year compared to men [1]. Older women, most especially those above the age of 80, have a significantly high incidence of stroke, and they are at an increased risk of having a stroke [5]. The prevalence and morbidity of stroke are also higher among women. Women are also less likely to recover from stroke, and they are also less likely to attend and gain support from stroke rehabilitation [2].

Socioeconomic-based criterion: Using the current status quo, acute stroke care for women generally cost more than men, which is not cost-effective for the government [7].

Ethics-based criterion: Older and pregnant women are vulnerable to stroke, and they have a disproportionately increased risk of having a stroke. Also, there is little and unequal public awareness and recognition of stroke among women [2].

Sustainability-based criterion: Using the current status quo will mean not achieving some of the 2030 SDG goals. This includes SDG 3, SDG 5 and SDG 10, and SDG 16 [12].

Summary

When comparing the argument for maintaining the status quo with the arguments against it, it is evident that there are more and stronger arguments against the status quo on the issue of stroke among Canadian women. The evidence-based criterion against the status quo is also stronger than the evidence-based criterion supporting it.

Alternative 1: increased public awareness of stroke symptoms among Canadian women

Arguments For

Evidence-based criterion: Increased public awareness and recognition of stroke in women is needed. This can be achieved through better and more up-to-date surveillance on stroke, better health policies, and more health promotion by the PHAC. This could potentially help reduce the incidence and prevalence of stroke among women, most especially older women. The greater public awareness will also enable stroke signs to be quickly recognized so that patients can be treated as quickly as possible [2]. This could also reduce the mortality and morbidity of stroke among women as it will improve their chances of survival and recovery and reduce the chance of having a disability (Table 1).

Socioeconomic-based criterion: Due to reduced morbidity that comes as a result of increased awareness and recognition of stroke in women, there will be fewer complications of a stroke in women, which means the lesser duration of time spent in hospitals which will reduce the cost of acute stroke care in women (Table 1).

Ethics-based criterion: Increased awareness will particularly be useful for older women who are vulnerable as well as at an increased risk of getting a stroke. Increased awareness and recognition of stroke in women will also help achieve gender equality and fairness (Table 1) [2].

Sustainable-based criterion: This will help promote the achievement of all four SDG goals that are related to the issue of stroke among Canadian women. This includes SDG 3, 5, 10 and 16 (Table 1) [12].

Arguments Against

Socioeconomic-based criterion: There will be increased costs associated with developing better and more up-to-date surveillance of stroke, developing new policies, and new health promotion strategies by the PHAC to minimize the issues regarding stroke among Canadian women.

Summary

Despite the increased cost of better surveillance, better health policies, and better health promotion on stroke among women, the benefits outweigh the cost as there are more and stronger arguments supporting this alternative.

Alternative 2: increased awareness of stroke risk factors among Canadian women

Arguments For

Evidence-based criterion: The increased awareness of stroke risk factors among women by the PHAC will help decrease stroke incidence among women, especially older women (Table 1) [2].

Socioeconomic-based criterion: Due to the increased awareness of stroke risk factors, the incidence and complications that occur as a result of stroke will be reduced, which will minimize the cost of acute stroke care (Table 1).

Ethics-based criterion: Vulnerable women such as the elderly and pregnant women should most especially be aware of stroke risk factors as this will enable them to prevent and reduce their risk of having a stroke (Table 1) [2].

Sustainable-based criterion: Increased awareness of stroke risk factors among women will help promote the attainment of SDG goals 3, 5, 10, and 16 (Table 1) [12].

Arguments Against

Socioeconomic-based criterion: There will be increased costs associated with promoting stroke risk factors among women.

Summary

Despite the increased cost of health promotion on stroke risk factors among women, the long-term advantages outweigh the disadvantages.

Alternative 3: improved stroke diagnosis among Canadian women

Arguments For

Evidence-based criterion: The increased use of MRI/CT imaging will improve stroke diagnosis among women [13]. This will also allow early and more accurate medical interventions to be made that reduce the incidence and morbidity of stroke among women (Table 1).

Ethics-based criterion: This will help promote equality and ensure that stroke diagnosis among women is just as accurate as men (Table 1).

Sustainable-based criterion: Improved stroke diagnosis among women will also help promote the attainment of SDG 3, 5, 10, and 16 (Table 1) [12].

Arguments Against

Socioeconomic-based criterion: Improving diagnosis of stroke in women using this approach will most likely require significant investments in new MRI/CT scans, which can be very expensive to buy and maintain [7]. Therefore, this approach is not cost-effective.

Summary

The high cost of purchasing new MRIs/CT scans is a significant disadvantage to this alternative. Although the initial cost will be high, the long-term benefits of improving stroke diagnosis and the diagnosis of other diseases are substantial. The benefits should eventually outweigh the cost over a long period of time.

Alternative 4: improved stroke recovery and rehabilitation among Canadian women

Arguments For

Evidence-based criterion: The increased motivation to undergo rehabilitation, as well as the increased family, peer, and public support for women undergoing long-term recovery and rehabilitation, could possibly improve their recovery and rehabilitation rates and possibly reduce stroke prevalence rate [3,4] (Table 1).

Ethics-based criterion: With improved motivation and support, more women will most likely recover from stroke, and more women will benefit from stroke rehabilitation just as much as men. This will help reduce the recovery and rehabilitation inequality between men and women [3,4] (Table 1). 

Sustainable-based criterion: Improved stroke recovery and rehabilitation among women will help contribute towards achieving SDG goals 3, 5, 10, and 16 [12] (Table 1).

Arguments Against

Socioeconomic-based criterion: The increased use of stroke rehabilitation and long-term care services will increase the long-term and total stroke cost.

Summary

Despite the increased cost of stroke rehabilitation, the potential long-term benefits of the services on the health outcomes of stroke patients are substantial, and it will eventually outweigh the cost as the chances of stroke reoccurring is also reduced due to rehabilitation.

Alternative 5: increased research on stroke among Canadian women

Arguments For

Evidence-based criterion: Increased research on stroke in women could reveal new risk factors, treatments, and rehabilitation strategies that will reduce the incidence, prevalence, morbidity, and mortality of stroke in women (Table 1).

Ethics-based criterion: Women that are susceptible to stroke, such as older women, will most especially benefit from increased research on stroke (Table 1).

Sustainable-based criterion: Increased research on stroke among women will help meet some of the requirements for SDG goals 3, 5, 10, and 16 (Table 1) [12].

Arguments Against

Socioeconomic-based criterion: Conducting research can be very expensive, and it will require a significant amount of money invested by the government. There is also no guarantee that the outcome of the research will be beneficial.

Summary

Despite the significant cost of conducting research as well as the uncertainty of outcomes until the research is conducted, the potential benefits are substantial and outweigh the cost. 

## Discussion

Recommendations on the alternatives and status quo

Based on the analysis of the supporting and opposing arguments using the evaluation criteria, it is recommended that the status quo be rejected and proceed with alternatives 1 and 2, which involve increased public awareness of stroke symptoms among Canadian women and increased public awareness of stroke risk factors among Canadian women. Applying these approaches could help reduce the incidence, prevalence, morbidity, and mortality of stroke among women in Canada. As shown in Table 1, the arguments supporting alternatives 1 and 2 fulfilled all four criteria, which include: 1) the evidence-based criteria, 2) the socioeconomic-based criteria, 3) the ethics-based criteria, and 4) the sustainable-based criteria. The status quo as well as alternatives 3, 4 and 5 do not fulfill all four evaluation criteria which explains why they are not recommended. Unlike alternatives 1 and 2, the status quo was only supported by the evidence-based criteria. Support from the other evaluation criteria are significantly lacking for the status quo. On the other hand, alternatives 3, 4 and 5 are lacking significant support from the socioeconomic-based evaluation criteria (Table 1).

Alternative 1, which involves the increased public awareness and recognition of stroke symptoms in women, is significantly needed. This could potentially help reduce the incidence of stroke among women, most especially older women. With reduced incidence, there will also be reduced prevalence of stroke among women. The greater public awareness will also enable stroke symptoms to be quickly recognized so that patients can be treated as quickly as possible. This could also reduce the mortality and morbidity of stroke among women as it will improve their chances of survival, and recovery and reduce the chances of having a disability. Due to the reduced morbidity that comes as a result of increased awareness and recognition of stroke symptoms in women, there will be less complications of stroke in women, which means lesser duration of time spent in hospitals reducing the cost of acute stroke care. Increased awareness will particularly be useful for older women and those that are vulnerable and are also at an increased risk of getting stroke. Increased awareness of stroke in women will also help achieve gender equality and fairness [2].

In addition to the increased public awareness of stroke symptoms among women in Canada, this article also recommends alternative 2 - the increased awareness of stroke risk factors among Canadian women. The increased awareness of stroke risk factors among women could help decrease stroke incidence among women, especially older women. Due to the increased awareness of stroke risk factors, the incidence and complications that occur as a result of stroke could be reduced which will minimize the cost of acute stroke care. Vulnerable women such as the elderly and pregnant women should especially be aware of stroke risk factors as this will enable them to prevent and reduce their risk of having stroke [2].

Limitations of the alternatives and status quo

One of the most important limitations to consider that was present in every opposing argument for each alternative presented in this article, including those that were recommended, is with regards to the feasibility of the approach. Each alternative including the status quo had at least one socioeconomic challenge that could potentially make it unfeasible. With regards to the status quo, the main socioeconomic challenge is that the current acute stroke care cost for women is generally greater than men, which is not a cost-effective trend for the government to sustain over a long period of time because as more women suffer from stroke on a yearly basis, the acute care cost for stroke will continue to increase at a higher rate if the cost are not reduced to level with men [7].

In alternative 1, increased public awareness and recognition of stroke symptoms among Canadian women, the main socioeconomic challenge that could potentially make it unfeasible is that there could be increased cost associated with the development of better surveillance, better health policies and better health promotion strategies by the PHAC to improve the public awareness and recognition of stroke symptoms among women in Canada [7].

In alternative 2, increased public awareness of stroke risk factors among Canadian women, socioeconomic challenges are similar to that of alternative 1 because it will most likely require increased cost and additional resources for the PHAC to develop health promotion strategies that focuses on improving the awareness of stroke risk factors among women [7].

The socioeconomic challenges in alternative 3 is significant because there are limited MRIs/CT scans in Canada [14]. This is supported by previous studies which have shown that the utilization of CT and MRI scanners in Canada is significantly less than other Organisation for Economic Co-operation and Development (OECD) countries [14]. Therefore, improving diagnosis of stroke in women will most likely require significant investments and purchase of new MRIs/CT scans to meet the objectives of this alternative which might not be feasible given the limited resources available by the Canadian provincial/territorial government to deliver universal health insurance to the population [7].

Alternative 4 is also potentially unfeasible because the increased use of stroke rehabilitation services will significantly increase the long-term and total cost of treating stroke [7].

Likewise, alternative 5 could also be unfeasible because conducting research can be very costly and there is no certainty and guarantee that the outcome of increased stroke research in women will eventually be beneficial when it comes to reducing the incidence, prevalence, morbidity, and mortality of stroke among Canadian women [7].

One way of overcoming these limitations is to encourage the federal government to provide more financial support for the PHAC. This will further ensure that the recommendations provided are fulfilled by the PHAC. 

## Conclusions

Upon analysis and discussion of the different alternative interventional strategies by using several evaluation criteria, the overarching recommendation of this article is for the Public Health Agency of Canada to develop improved health promotional strategies on the issue of stroke among women in Canada. This could assist in reducing the incidence, prevalence, morbidity, and mortality of stroke among Canadian women. This can be achieved using two approaches and alternative interventional strategies: 1) increased public awareness of stroke symptoms among Canadian women, and 2) increased public awareness of stroke risk factors among Canadian women. Taken together, by accepting and applying these recommendations regarding the alternative interventional strategies, not only could the incidence, prevalence, morbidity, and mortality of stroke among Canadian women be reduced, but it will also enable the federal government to meet some of the UN 2030 sustainable development goals such as SDG 3, SDG 5, SDG 10 and SDG 16. 
